# Bridging of Finger-Prick Volumetric Absorptive Microsampling and Conventional Venous Sampling with Clinical Specimens for Direct Oral Anticoagulants Analysis by LC–MS/MS

**DOI:** 10.21627/bhyw5v67

**Published:** 2026-07-08

**Authors:** Yu-Fong Peng, Sung-Chun Tang, Ching-Hua Kuo, Shin-Yi Lin

**Affiliations:** School of Pharmacy, College of Medicine, National Taiwan University, Taipei 100, Taiwan; The Metabolomics Core Laboratory, NTU Centers of Genomic and Precision Medicine, National Taiwan University, Taipei 100, Taiwan; Stroke Center and Department of Neurology, National Taiwan University Hospital, Taipei 100, Taiwan; School of Pharmacy, College of Medicine, National Taiwan University, Taipei 100, Taiwan; The Metabolomics Core Laboratory, NTU Centers of Genomic and Precision Medicine, National Taiwan University, Taipei 100, Taiwan; Department of Pharmacy, National Taiwan University Hospital, Taipei 100, Taiwan; School of Pharmacy, College of Medicine, National Taiwan University, Taipei 100, Taiwan; Department of Pharmacy, National Taiwan University Hospital, Taipei 100, Taiwan

**Keywords:** Conversion factor, direct oral anticoagulants, liquid chromatography–tandem mass spectrometry, finger-prick blood, plasma, volumetric absorptive microsampling

## Abstract

OBJECTIVES: Direct oral anticoagulants (DOACs) are the first-line therapy for stroke prevention in non-valvular atrial fibrillation. Exposure-response analysis has shown correlations between drug concentrations and clinical outcomes, making therapeutic drug monitoring a valuable tool. Liquid chromatography–tandem mass spectrometry (LC–MS/MS) enables sensitive and accurate quantification for clinical analysis. Volumetric absorptive microsampling (VAMS) is a minimally invasive technique for collecting dried blood specimens. However, the translation from conventional venous blood sampling to VAMS and the conversion between plasma and whole-blood concentrations remain unclear. METHODS: We developed an LC–MS/MS method for quantifying four DOACs—dabigatran, apixaban, rivaroxaban, and edoxaban—in VAMS samples and applied it to paired clinical specimens to compare venous and finger-prick blood and establish conversion factors. RESULTS: Validation results support accuracy and precision of VAMS analysis by LC–MS/MS. Comparative analysis demonstrated no significant differences in concentrations between finger-prick and venous blood for dabigatran, apixaban, and rivaroxaban. Using paired clinical samples, conversion factors were derived via weighted Deming regression: 1.88, 1.57, 1.64, and 1.08 for dabigatran (*n* = 30), rivaroxaban (*n* = 33), apixaban (*n* = 35), and edoxaban (*n* = 36), respectively. The hematocrit effect was statistically significant for dabigatran. Bland–Altman analysis showed that more than 80% of samples fell within ±20% of the mean between estimated and measured plasma concentrations. CONCLUSION: These findings support the potential clinical utility of VAMS with LC–MS/MS as an accurate and convenient tool for DOAC monitoring, facilitating future implementation of precision medicine in anticoagulation management to minimize bleeding and stroke events.

## Non-Specialist Summary

Doctors use blood thinners to prevent strokes in people with irregular heartbeats. This study tested a new, finger-prick blood sampling method (volumetric absorptive microsampling, VAMS) with a highly sensitive lab test to measure levels of four common blood thinners. We demonstrated that concentrations measured from VAMS samples can be reliably converted to standard vein blood concentrations. This approach could make drug level monitoring easier, helping tailor doses and reduce risks of bleeding or stroke.

## 1. Introduction

Atrial fibrillation (AF) is the most common cardiac arrhythmia worldwide and is associated with an increased risk of thromboembolism and embolic stroke [1, 2]. Clinical guidelines emphasize the importance of stroke prevention and recommend the use of oral anticoagulants in high-risk patients [3]. Direct oral anticoagulants (DOACs) have become the preferred treatment over warfarin in patients with non-valvular AF [4]. Randomized controlled trials have demonstrated that DOACs are non-inferior to warfarin in preventing stroke and systemic embolism while significantly reducing the risk of major bleeding, particularly intracranial hemorrhage (ICH) [5]. Moreover, compared with warfarin, DOACs offer more predictable pharmacokinetics and fewer interactions with food and other medications [3, 6].

Despite these advantages, treatment with DOACs is not free from complications. According to the real-world claims data in Taiwan, the incidence rate is 2.58 events per 100 person-years for major bleeding and 0.40 for ICH [7]. Several studies have shown that bleeding risk and stroke are correlated with plasma concentrations of DOACs [8–22]. Increasing evidence supports therapeutic drug monitoring (TDM) as a valuable strategy to optimize stroke prevention while minimizing bleeding risk, particularly in vulnerable populations such as the elderly, patients with renal impairment, and those with a history of stroke [8–10, 12, 13, 16–18, 21, 23].

Coagulation assays, such as heparin-calibrated anti-Xa, are widely used to assess plasma DOAC concentrations, particularly in emergency settings. Although they offer rapid turnaround, their accuracy and precision are limited, especially at low and high concentrations [24–26]. In contrast, liquid chromatography–tandem mass spectrometry (LC–MS/MS) is the gold standard for quantification, providing highly accurate DOAC measurements across a wide calibration range, and is well suited for TDM. Previous LC–MS/MS methods have primarily been applied to measure DOAC concentrations in plasma. However, plasma collection by conventional venous sampling (CVS) requires laboratory infrastructure for proper handling and preservation, restricting the clinical utility of LC–MS/MS. Consequently, alternative sampling strategies are needed to enable broader application of this method.

Volumetric absorptive microsampling (VAMS) is a novel technique that enables collection of dried specimens of capillary blood via finger prick using a lancet, featuring a calibrated polymeric tip [27]. Similar to dried blood spot (DBS) sampling, VAMS offers multiple advantages over CVS, including minimal invasiveness, reduced biohazard risk, simplified storage and transport, and improved drug stability at room temperature (RT) [28–30]. Additionally, unlike DBS, VAMS collects a fixed volume of blood independent of hematocrit (HCT) values, eliminating the need for blood volume correction and thereby reducing analytical variability [27, 28, 31]. These features make VAMS particularly attractive for remote or patient-driven sample collection. However, the implementation of VAMS in clinical practice requires the establishment of reliable conversion factors for translating finger-prick VAMS concentrations into CVS-equivalent values [32–34]. Establishing these factors is essential because most clinical studies have used CVS for drug monitoring, and therapeutic ranges for the majority of drugs are defined in plasma, serum, or venous blood [35]. Such quantitative relationships have been established for several drugs with acceptable predictive performance [34]. The utility of microsampling techniques, including VAMS, for DOAC LC–MS/MS analysis has been demonstrated in several studies [26, 29–31, 36–38]. However, key limitations remain. Notably, analyte stability on VAMS devices beyond 30 days, which is critical for remote or delayed sample analysis, has not been evaluated. Furthermore, supporting evidence from real-world patient specimens remains limited, leaving the clinical applicability of these microsampling strategies uncertain. With the growing attention on DOAC TDM for improving safety and efficacy, an effective and reliable VAMS sampling strategy may help realize the potential of precision therapy for DOACs.

In this study, all four DOACs approved by both the US Food and Drug Administration (FDA) and the European Medicines Agency (EMA)—dabigatran, apixaban, rivaroxaban, and edoxaban—were included to ensure broad applicability across patients at risk of bleeding events. An LC–MS/MS method was developed and validated to quantify the concentrations of four DOACs using VAMS devices. For potential DOAC concentration differences in finger-prick blood and venous blood, paired samples were collected from patients undergoing DOAC therapy. We also collected paired finger-prick blood and plasma samples to establish conversion factors between VAMS-based finger-prick and CVS-based plasma concentrations. By using samples from individuals undergoing DOACs therapy, this study provides the first real-world data for all FDA- and EMA-approved DOACs, supporting the accuracy of VAMS and highlighting potential issues when applying this sampling strategy in clinical care.

## 2. Materials and Methods

### 2.1. Chemicals and Materials

Dabigatran, apixaban, [^13^C, *d*_3_]-apixaban, rivaroxaban, edoxaban, and [*d*_6_]-edoxaban were purchased from Toronto Research Chemicals (Toronto, ON, Canada). [^13^C_6_]-Dabigatran and [^13^C_6_]-rivaroxaban were purchased from Alsachim (Illkirch-Graffenstaden, France). LC–MS-grade acetonitrile and isopropanol were obtained from J.T. Baker (Phillipsburg, NJ, USA). MS-grade methanol was purchased from Scharlau Chemie (Sentmenat, Barcelona, Spain). MS-grade formic acid was obtained from Sigma-Aldrich (St. Louis, MO, USA). MS-grade ammonium acetate was purchased from Merck (Darmstadt, Germany). Twenty microliter Mitra^®^ clamshells for VAMS were purchased from Neoteryx (Torrance, CA, USA). Stock solutions of dabigatran and [^13^C_6_]-dabigatran, each at 1.0 mg mL^−1^, were prepared in methanol/water (9:1) containing 0.1% (v/v) formic acid. Apixaban and [^13^C, *d*_3_]-apixaban were prepared in methanol at 1.0 mg mL^−1^ and 0.5 mg mL^−1^, respectively. Rivaroxaban and [^13^C_6_]-rivaroxaban were prepared in acetonitrile/water (9:1) at 1.0 mg mL^−1^ and 0.1 mg mL^−1^, respectively. Edoxaban and [*d*_6_]-edoxaban were prepared in acetonitrile/water (3:2) at 1.0 mg mL^−1^. The mixed working solutions of dabigatran, rivaroxaban, apixaban, and edoxaban were prepared at 100 µg mL^−1^ by diluting the stock solution with methanol. All of the solutions were stored at −20 °C.

### 2.2. LC–MS/MS System

The LC separations were performed using an Agilent 1290 UHPLC system equipped with a binary solvent pump, an autosampler, a sample reservoir, and a column oven (Agilent Technologies, Waldbronn, Germany). The coupled mass spectrometer was an Agilent 6460 triple-quadrupole system (Agilent Technologies, Waldbronn, Germany). A Kinetex reversed-phase core–shell C18 column (2.1 × 50 mm, 2.6 μm, 100 Å, Phenomenex, Torrance, CA, USA) was used. The mobile phase consisted of 0.1% formic acid and 10 mM ammonium acetate in water (solvent A) and 0.1% formic acid and 10 mM ammonium acetate in isopropanol-acetonitrile (9:1, v/v) (solvent B). The flow rate was 0.35 mL min^−1^. The total chromatographic run time was 5.7 min. The gradient profile began at 0% B for 0.5 min, increased to 6% B over 0.1 min, was maintained at 6% B for 0.6 min; was raised to 25% B over 0.5 min, to 27.5% B over 0.5 min, and to 50% B over an additional 0.5 min; and was then maintained at 50% B for 1 min. Finally, the column was re-equilibrated to 0% B for 2 min until the next injection. The temperature of the sample reservoir was maintained at 4 °C, and the column oven was set at 55 °C. The injection volume was 3 μL.

Positive electrospray ionization mode was utilized with the following parameters: 350 °C dry gas temperature, 10 L min^−1^ dry gas flow rate, 45 psi nebulizer pressure, 350 °C sheath gas temperature, 11 L min^−1^ sheath gas flow rate, 3500 V capillary voltage, and 500 V nozzle voltage. MS data acquisition was executed in multiple reaction monitoring mode, and the mass transitions were 472.2→289, 472.2→144 for dabigatran, 478.2→295.1, 478.2→144 for [^13^C_6_]-dabigatran, 436.1→144.9, 436.1→72.9 for rivaroxaban, 442.1→144.9, 442.1→72.9 for [^13^C_6_]-rivaroxaban, 460.2→443.1, 460.2→199 for apixaban, 464.2→447.1, 464.2→203.1 for [^13^C, *d*_3_]-apixaban, 548.1→366.1, 548.1→152 for edoxaban, 554.1→372.1, 554.1→158.1 for [*d*_6_]-edoxaban.

### 2.3. Sample Preparation and Extraction

To prepare dried blood samples using VAMS, the VAMS tip was held at a 45-degree angle in whole blood until fully saturated, followed by a brief additional equilibration period. The tip was then dried at room temperature overnight. After drying, the VAMS device was stored in a sealed plastic bag with desiccant or in an electronic humidity-controlled cabinet until extraction.

Before VAMS extraction, the VAMS tip was transferred into an Eppendorf tube. Three hundred microliters of water containing 0.1% (v/v) formic acid and 10 ng mL^−1^ internal standards (ISs) was added, and the samples were extracted using a Geno/Grinder 2010 (SPEX^®^ Sample Prep, Metuchen, NJ) for 2 min at 1000 rpm. A 700 µL aliquot of acetonitrile was then added to the formic acid extract and extracted using the Geno/Grinder 2010 for an additional 3 min. After centrifugation at 18,000 rcf (relative centrifugal force) for 5 min, 800 μL of supernatant was placed into another Eppendorf tube and evaporated using a centrifugal vaporizer (Thermo SpeedVac^®^ SPD111V, Waltham, MA).

For plasma sample extraction, 100 μL of plasma was extracted with 800 μL of methanol containing 2 ng mL^−1^ IS by shaking for 2 min at 1000 rpm using a Geno/Grinder 2010. The extract was then centrifuged at 15,000 rcf for 5 min. Four hundred microliters of supernatant was transferred to a new Eppendorf tube. The plasma extracts were dried with a centrifugal vaporizer.

Before LC–MS/MS analysis, the VAMS and plasma residues were reconstituted with 200 μL of methanol and filtered through a 0.2 μm polypropylene (PP) membrane filter (RC-4, Sartorius, Göttingen, Germany).

### 2.4. Method Validation

Full method validation was conducted according to ICH guideline M10 on bioanalytical method validation and study sample analysis. Whole blood samples from healthy volunteers were collected in K_2_EDTA tubes (BD Vacutainer^®^) through venous puncturing. To prepare drug-spiked VAMS samples for establishing the calibration curve, 5 μL of DOAC working solution ranging from 0.1 to 20 μg mL^−1^ was separately spiked into 95 μL of whole blood and then allowed to equilibrate for 30 min at RT before being absorbed by a VAMS tip. The VAMS samples were dried at RT in the dark overnight. Quality control (QC) samples were prepared using a similar protocol as the calibration-curve samples, except a working solution with a concentration ranging from 0.2 to 30 μg mL^−1^ was used. For QC samples controlling HCT, before drug spiking, whole blood samples were centrifuged to separate plasma and the plasma was transferred to obtain HCT values of 20%, 40%, and 60%. The concentrations of the lower limit of quantification (LLOQ), low, medium, high, and upper limit of quantification (ULOQ) QC samples were 5, 10, 100, 750, and 1000 ng mL^−1^, respectively.

Sensitivity was defined as the LLOQ. Selectivity was evaluated using six blank matrices extracted with solvents without an IS. Carry-over was determined by injecting blank methanol after the ULOQ sample. The analyte peak areas should be less than 20% of those of LLOQ samples and less than 5% of those for ISs.

Calibration curves were assessed from LLOQ to ULOQ, including seven-point levels at the following concentrations: 5, 10, 100, 250, 500, 750, and 1000 ng mL^−1^. The four calibration curves were obtained using linear regression of the area ratio of target analytes to corresponding ISs versus the target analyte concentration with 1/*x* weighting factors.

Four concentration levels (LLOQ, low, medium, and high) QC samples were used to evaluate accuracies, precision, recoveries, and matrix factors (MFs). Intra-day accuracy and precision were determined by analyzing five different QC samples per concentration within the same day. For inter-day accuracy and precision, 15 different samples per concentration were analyzed at 3 separate days (five QC samples per day). The results for intra-day and inter-day precision were determined as coefficients of variation (CV, %), and intra- and inter-day accuracies were evaluated by calculating the percentages of the nominal values and their SDs. Three replicates of pre-spiked samples (DOACs were added to blank blood samples before extraction), post-spiked samples (DOACs were added to the processed blank samples), and standard samples (DOACs were added to the processed methanol solutions) were used to evaluate recoveries and MFs for each concentration level. Recoveries were calculated by comparing the peak areas of the pre-spiked samples with the peak area of the post-spiked samples. MFs were calculated by comparing the peak areas of the post-spiked samples with the peak area of the standard samples. Both expected ratios and SDs were calculated.

Matrix effects were assessed with three replicates of low- and high-level QC samples from six sources and were evaluated by calculating the percentages of the nominal values and their SDs. HCT-related bias for recoveries, MFs, and accuracies was evaluated using low- and high-level QC samples with HCT values of 20%, 40%, and 60%. Three replicates were utilized for each condition, and the calculation was identical to that described in the preceding paragraph.

Dilution integrity was assessed for one dilution factor: twofold dilution of 1500 ng mL^−1^ (1.5 ULOQ) samples by methanol and blank matrix. Five replicates were utilized, and the accuracies and SDs were calculated.

Stability tests on the VAMS device were conducted using low- and high-level QC samples stored under three sets of conditions (RT, 4 °C, and −20 °C) for 7, 30, and 120 days. Three replicates were utilized per temperature, duration, and concentration. The stabilities under each set of conditions were evaluated by quantification accuracies compared to freshly prepared QC samples (*t* = 0) and their SDs. Long-term (120 days) recoveries were evaluated using three replicates of medium-level QC samples at RT.

### 2.5. Collection of the Clinical Samples

All clinical VAMS and plasma samples were collected at National Taiwan University Hospital. These samples were used to compare finger-prick blood with venous blood and to establish the correlation between VAMS-derived and plasma concentrations. Ethics committee approval was obtained from the Research Ethics Committee of the National Taiwan University Hospital (Rec. No. 202106062RINB). This study was registered in the clinicaltrials.gov database (NCT05333666). All patients who participated in this study signed informed consent statements prior to enrollment. A total of 5 mL of blood was collected in the K_2_EDTA tubes (BD Vacutainer^®^) through venous puncturing. Plasma samples were obtained by centrifugation at 3000 rcf for 15 min and stored at −80 °C. VAMS samples were collected through finger pricking at the same time of venous puncturing. Additionally, another VAMS sample was prepared using blood drops from each venous sample.

### 2.6. Data Analysis

Agilent MassHunter Quantitative Analysis 10.0 (Agilent Technologies, Waldbronn, Germany) was used to compute DOAC concentrations. Validation data were processed with the AnalyticalMethodValidation.jl package [39]. Paired *t*-tests and ordinary linear regression were performed using HypothesisTests.jl [40] and GLM.jl [41], respectively. Conversion of DOAC concentration was processed by MethodComparisonRegression.jl [42]. Four conversion methods were included (i.e., mean of ratios, ratio of means, Deming regression, and weighted Deming regression); they were compared using three performance metrics (i.e., mean percentage error [MPE], root mean squared percentage error [RMSPE], and mean absolute percentage error [MAPE]) and estimated by leave-one-out cross-validation (LOOCV). Bland–Altman analysis was used for prediction performance. All plots were generated using MethodComparisonRegression.jl.

## 3. Results

### 3.1. Method Validation of LC–MS/MS Analysis for DOAC Quantification in VAMS

We adapted a previously validated DBS protocol for use with VAMS to quantify the four DOACs [30]. The original DBS protocol involved pre-rinsing the sampling device with 300 μL of 0.1% formic acid for improvement of recovery and extraction with 700 μL of acetonitrile. This procedure was directly tested on VAMS and compared against alternative protocols using deionized water as the rinsing solution and methanol as the extraction solvent. All tested protocols yielded recoveries greater than 90%; thus, the original DBS protocol was selected.

Method validation was performed using drug-spiked VAMS samples, which were analyzed by LC–MS/MS after extraction. Selectivity was assessed using six independent blank samples, and all sample areas met the criteria (20% of LLOQ for target analytes; 5% for IS). Linearity was evaluated over the concentration range 5−1000 ng mL^−1^, with the coefficients of determination (*R*^2^) values exceeding 0.99 for all four DOACs (Table 1). The LLOQ was established at 5 ng mL^−1^, offering sufficient sensitivity for quantifying drugs at the lower levels of the reported therapeutic range [43]. Carry-over was assessed with an injection of methanol after the ULOQ sample, and the area met the criteria (20% of LLOQ for target analytes; 5% for IS).

**Table 1.** Calibration curves for quantification of four DOACs in VAMS samples.

Analyte	Range (ng mL^−1^)	Calibration curve	*R* ^2^
Dabigatran	5–1000	*y* = 0.0192*x* − 0.0255	0.990
Rivaroxaban	5–1000	*y* = 0.0336*x* − 0.0185	0.997
Apixaban	5–1000	*y* = 0.0239*x* − 0.0321	0.998
Edoxaban	5–1000	*y* = 0.0232*x* − 0.0179	0.998

Accuracy, precision, recovery, and MFs were evaluated at four concentration levels: LLOQ, low, medium, and high (Table 2). Recoveries ranged from 96.2% to 114.8%; MFs ranged from 123.3% to 135.4% for dabigatran and from 91.1% to 116.8% for the other three DOACs. Isotope-labeled ISs effectively corrected for MFs and processing errors, and the resulting quantification accuracies fell within acceptable limits at all tested levels. Repeatability (intra-day precision) and intermediate precision (inter-day precision) were generally less than 10%, confirming both robustness and reproducibility.

**Table 2.** Accuracy, precision, recovery, and MF for quantification of four DOACs in VAMS samples.

Analyte	Test Level^a^	Intra-day (*n* = 5)^b^		Inter-day (*n* = 15)^b^		Recovery (*n* = 3)^b^ ($\bar{X}$ ± SD%)	MF (*n* = 3)^b^ ($\bar{X}$ ± SD%)
		Accuracy ($\bar{X}$ ± SD%)	Precision (CV%)	Accuracy ($\bar{X}$ ± SD%)	Precision (CV%)		
Dabigatran	LLOQ	118.3 ± 0.6	3.1	116.4 ± 3.6	3.1	107.9 ± 2.8	135.4 ± 9.9
	Low	103.3 ± 3.5	5.7	107.5 ± 6.2	7.1	106.5 ± 5.3	134.1 ± 15.1
	Medium	94.8 ± 8.2	5.8	98.6 ± 5.7	6.5	109.9 ± 4.3	130.6 ± 4.9
	High	100.8 ± 4.8	6.0	102.5 ± 6.1	6.0	99.1 ± 7.5	123.3 ± 3.7
Rivaroxaban	LLOQ	118.2 ± 7.0	6.1	118.8 ± 7.3	6.8	114.8 ± 7.7	103.1 ± 16.4
	Low	100.3 ± 3.3	5.4	106.2 ± 5.7	6.9	107.6 ± 5.6	94.4 ± 10.0
	Medium	103.6 ± 10.4	7.0	110.4 ± 7.8	8.4	111.3 ± 5.4	97.0 ± 3.0
	High	102.3 ± 3.6	4.0	101.8 ± 4.1	4.0	99.0 ± 5.2	92.9 ± 2.5
Apixaban	LLOQ	119.8 ± 1.6	2.6	121.3 ± 3.1	2.6	108.5 ± 3.0	95.1 ± 4.4
	Low	104.0 ± 2.8	5.0	108.8 ± 5.5	7.3	108.4 ± 5.1	91.9 ± 2.4
	Medium	96.1 ± 7.9	5.9	104.9 ± 6.2	10.3	111.9 ± 5.0	95.3 ± 2.1
	High	112.3 ± 4.1	4.2	112.5 ± 4.8	4.2	100.4 ± 5.9	91.1 ± 1.8
Edoxaban	LLOQ	114.8 ± 7.4	5.6	117.6 ± 6.6	7.4	102.4 ± 7.6	116.8 ± 14.1
	Low	111.1 ± 4.3	6.1	106.9 ± 6.5	7.2	103.7 ± 5.3	102.0 ± 12.0
	Medium	101.3 ± 8.9	6.1	106.5 ± 6.9	7.2	107.7 ± 4.8	107.8 ± 4.0
	High	104.1 ± 5.5	5.1	102.2 ± 5.2	5.1	96.2 ± 6.0	102.1 ± 2.9

a LLOQ, 5 ng mL^−1^; Low, 10 ng mL^−1^; Medium, 100 ng mL^−1^; High: 750 ng mL^−1^.

b Intra-day validation was evaluated with five replicates for each level; inter-day validation was evaluated in 3 days and five replicates/day for each level (i.e., a total of 15 replicates); recovery and MF were evaluated with three replicates for each level and sample type.

As our calibration range exceeded the higher levels of the reported therapeutic range [43], we only evaluated dilution integrity at one concentration level (1500 ng mL^−1^) and one dilution factor (twofold) (Table S1). All accuracies were within 95% to 105%, supporting the use of twofold dilution for samples with a concentration slightly higher than 1000 ng mL^−1^.

Matrix effects were evaluated using samples from six sources, and the accuracies were within 95% to 105% for both low- and high-level samples (Table S2). The effects of HCT on recovery, MF, and accuracy were evaluated at 20%, 40%, and 60% (Table S3). For all four DOACs, there were no HCT effects on recoveries (89.9% to 117.1%); however, effects on the MFs were observed for edoxaban. Although MFs of high-level QC samples increased from 113.0% to 135.6% for edoxaban, the effectiveness of IS calibration gave quantification accuracies that remained within acceptable limits at all tested levels and for all sample sources and HCT values.

The stability of DOACs on the VAMS device was assessed at 7, 30, and 120 days under three different temperature conditions (RT, 4 °C, and −20 °C) and two concentration levels (low and high). Across all conditions and time points, the quantification accuracy ranged from 89.5% to 114.2% compared to *t* = 0 samples (Table 3). In long-term testing at 120 days, recoveries for medium-level QC samples stored at RT ranged from 92.4% to 116.5%. These findings indicate that VAMS provides adequate stability for four DOACs for 120 days.

**Table 3.** Stability (quantification accuracy compared to *t* = 0 samples) on the VAMS device for four DOACs.

Test level^a^	Condition	Day	Apixaban ($\bar{X}$ ± SD%)	Edoxaban ($\bar{X}\pm \text{SD\%}$)	Dabigatran ($\bar{X}\pm \text{SD\%}$)	Rivaroxaban ($\bar{X}\pm \text{SD\%}$)
Low (*n* = 3)	RT^b^	7	97.7 ± 5.4	99.2 ± 8.9	99.1 ± 4.2	103.7 ± 5.5
		30	99.9 ± 4.4	105.0 ± 6.8	101.4 ± 5.5	105.0 ± 4.5
		120	111.7 ± 8.7	96.6 ± 8.7	107.1 ± 6.4	114.2 ± 11.5
	4 °C	7	102.0 ± 4.4	107.1 ± 6.5	99.3 ± 4.6	107.7 ± 7.8
		30	99.1 ± 4.6	105.4 ± 5.8	100.9 ± 5.2	104.9 ± 4.0
		120	107.7 ± 7.4	103.2 ± 6.4	105.8 ± 6.0	108.4 ± 7.9
	−20 °C	7	101.6 ± 5.0	107 ± 5.5	99.9 ± 3.8	108.5 ± 11.0
		30	98.1 ± 4.0	103.7 ± 6.9	99.7 ± 4.6	106.2 ± 7.8
		120	104.2 ± 8.2	98.8 ± 10.8	102.8 ± 7.8	107.1 ± 10.6
High (*n* = 3)	RT^b^	7	98.7 ± 5.5	95.7 ± 7.5	98.6 ± 4.9	97.7 ± 5.3
		30	99.9 ± 8.4	94.2 ± 8.8	105.7 ± 11.3	101.5 ± 9.7
		120	91.0 ± 4.0	97.0 ± 6.0	109.1 ± 6.7	102.6 ± 5.0
	4 °C	7	103.8 ± 4.4	100.6 ± 5.6	103.7 ± 5.3	102.4 ± 5.1
		30	102.32 ± 4.3	100.7 ± 6.5	110.7 ± 8.9	104.4 ± 5.9
		120	89.5 ± 4.0	97.2 ± 6.6	106.4 ± 7.2	100.4 ± 5.3
	−20 °C	7	103.6 ± 5.4	99.8 ± 7.2	103.5 ± 5.9	102.3 ± 4.8
		30	98.6 ± 4.2	94.5 ± 5.7	105.0 ± 6.2	98.9 ± 4.6
		120	89.9 ± 4.1	97.5 ± 6.2	109.4 ± 6.8	101.7 ± 5.2

a Low, 10 ng mL^−1^; High, 750 ng mL^−1^; the stability was evaluated with three replicates for each level.

b RT, room temperature.

### 3.2. Comparison of DOAC Concentration in Finger-Prick Blood and Venous Blood

To assess potential differences in drug concentrations in finger-prick blood and venous blood, paired VAMS samples of finger-prick blood and CVS-collected venous blood were compared (*n* = 72). Paired *t*-tests showed that the mean differences were less than 2 ng mL^−1^ for both apixaban and rivaroxaban, less than 5 ng mL^−1^ for dabigatran, and 7.75 ng mL^−1^ for edoxaban, the latter being statistically significant (*p* = 0.0061; Table 4). Scatter plots (Figure S1) between finger-prick blood and venous blood samples showed high correlation, with *R*^2^ values of 0.91 for dabigatran (*n* = 11), 0.98 for apixaban (*n* = 19), 0.99 for rivaroxaban (*n* = 21), and 0.92 for edoxaban (*n* = 21). Bland–Altman analysis (Figure S2) showed that 81.8% of dabigatran, 88.9% of apixaban, and 85.7% of rivaroxaban samples fell within ±20% of the mean between finger-prick blood and venous blood measurements. For edoxaban, because of significant concentration differences, a conversion factor estimated by weighted Deming regression was applied, resulting in agreement of 76.2% within the ±20% range.

**Table 4.** Paired *t*-tests comparing finger-prick blood samples to venous blood samples.

Analyte	Mean difference (ng mL^−1^)	*p*-Value
Dabigatran	−4.45	0.2841
Rivaroxaban	−1.36	0.2487
Apixaban	−1.54	0.4293
Edoxaban	7.75	0.0061

### 3.3. Establishment of Conversion Factors from VAMS to Plasma Concentrations

To derive conversion factors between DOAC concentrations in whole blood and plasma, paired finger-prick VAMS samples and CVS-collected plasma samples were collected from patients (*n* = 134). Scatter plots (Figure 1) show strong linearity between VAMS and plasma concentrations, with *R*^2^ values of 0.96 for dabigatran (*n* = 30), 0.93 for apixaban (*n* = 35), 0.99 for rivaroxaban (*n* = 33), and 0.96 for edoxaban (*n* = 36). The regression slopes are significantly greater than one, and the intercepts are not statistically different from zero (Table S4), indicating preferential distribution of DOACs in plasma over erythrocytes.

**Figure 1.**
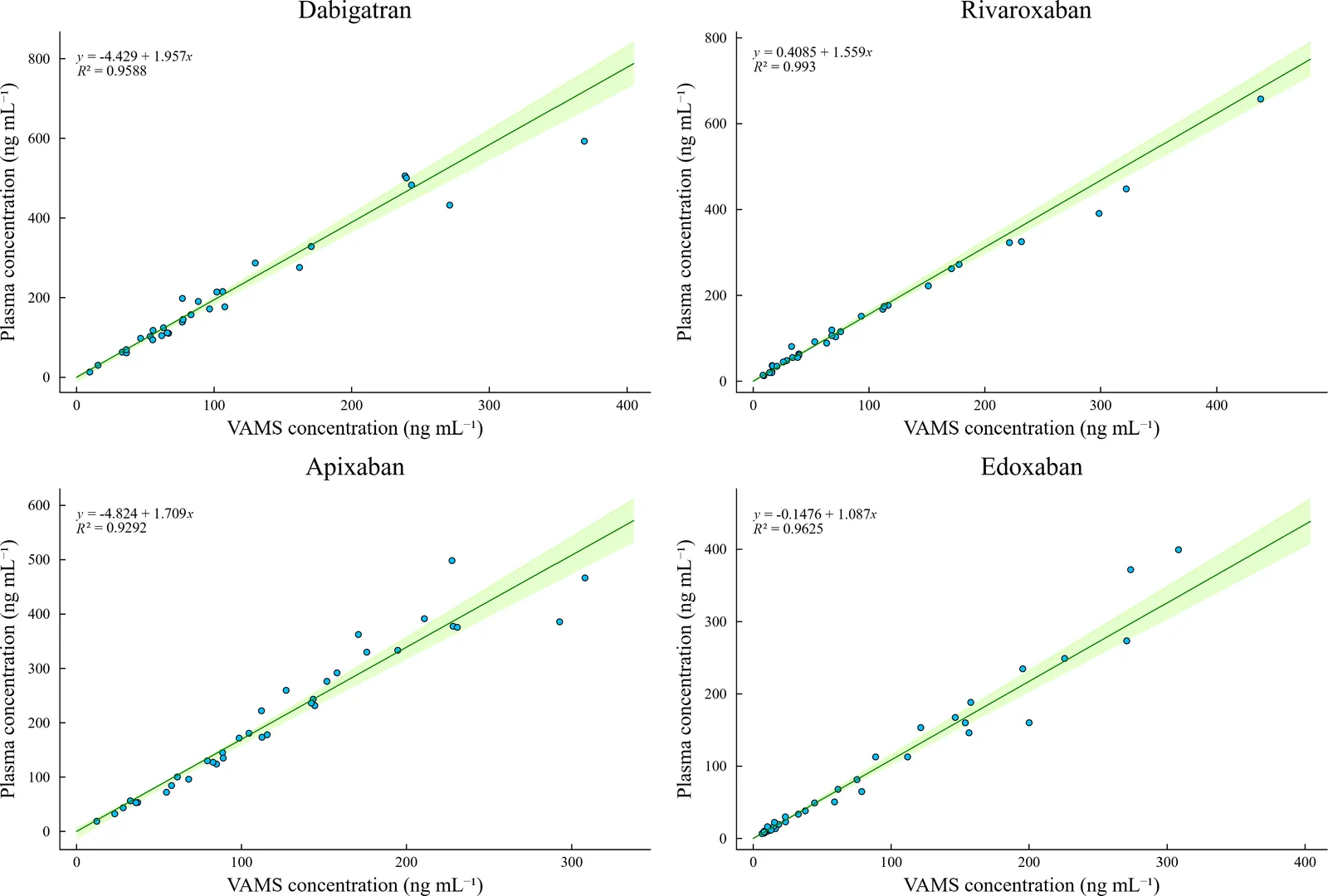
Correlation between DOAC concentrations quantified in paired finger-prick VAMS and plasma samples. Light-green regions are 95% confidence intervals. The coefficients of determination (*R*^2^) and weighted Deming regression formula are indicated.

To identify the optimal method for estimating plasma concentrations from VAMS, we compared four approaches: mean of ratios, ratio of means, Deming regression (zero intercept), and weighted Deming regression (zero intercept). LOOCV was used for method comparison, with MPE, RMSPE, and MAPE as performance metrics (Table S5). The MPE values were all very close to 0, with most of them less than 5%, indicating minimal systematic bias in the conversion estimates. For edoxaban, the errors were slightly larger (RMSPE ~ 16%, MAPE ~ 13%); the other three DOACs showed RMSPEs of less than 15% and MAPEs of approximately 10%.

Among all the methods tested, unweighted Deming regression performed the worst, mean of ratios performed slightly better than ratio of means, and weighted Deming regression produced the lowest MPE and RMSPE across the four DOACs. Because weighted Deming regression performed the best and adhered more closely to statistical assumptions, it was selected as the final conversion method, yielding conversion factors of 1.88 for dabigatran, 1.57 for rivaroxaban, 1.64 for apixaban, and 1.08 for edoxaban.

### 3.4. Performance Evaluation of Plasma Concentration Estimation Using VAMS

To assess the predictive accuracy of these conversion factors, we carried out Bland–Altman analysis comparing measured plasma concentrations and VAMS-estimated plasma concentrations (Figure 2). The results indicate that 93.3% of dabigatran, 87.9% of rivaroxaban, 85.7% of apixaban, and 80.6% of edoxaban samples exhibited less than 20% deviation between measured and estimated plasma concentrations relative to the means. Additional predictive metrics (Table S6) showed MPEs near zero, RMSPEs less than 15%, and MAPEs less than 10% for all of the DOACs except edoxaban. These results confirm the accuracy and robustness of the conversion models.

**Figure 2.**
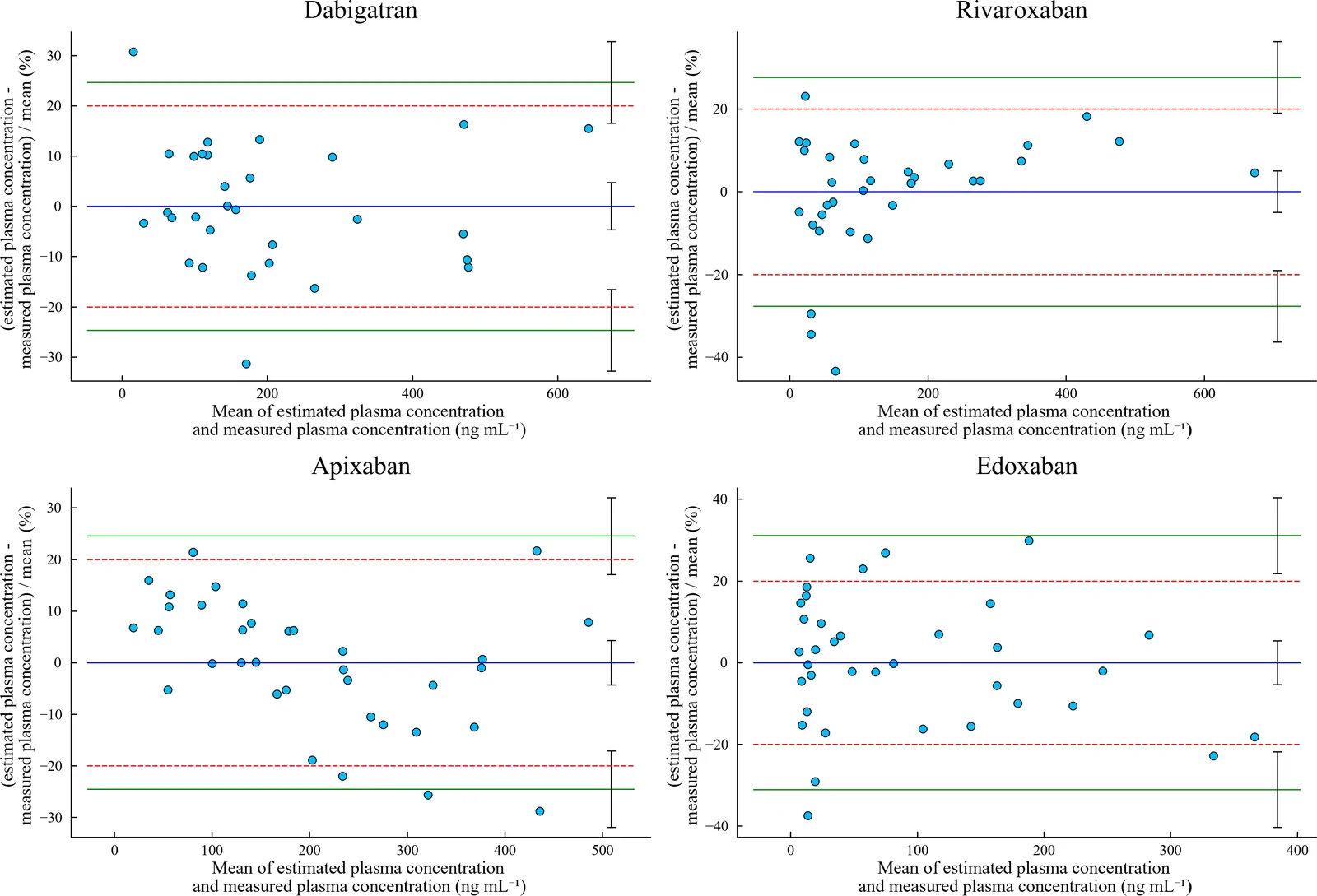
Bland–Altman plots for DOAC concentrations showing the percentage deviations between measured and estimated plasma concentration. The mean bias (blue line), upper and lower 95% limit of agreements (green lines), 20% estimation error acceptance criteria (dashed red lines), and 95% confidence intervals (black error bars) are indicated.

Given the heterogeneous distribution of DOACs between plasma and erythrocytes, HCT levels may influence the fraction of drug present in plasma and thereby affect the estimation accuracy. Figure S3 illustrates the correlation between HCT and the percentage difference between measured and estimated plasma concentrations. For dabigatran, a significant correlation was observed (*p* = 0.0058), whereas no significant correlations were found for other DOACs. Nonetheless, the associated prediction errors remained less than 20% and the coefficient of determination was only 0.34.

## 4. Discussion

In recent years, dried microsampling technologies have revolutionized the field of bioanalysis for their advantages of minimal invasiveness, easy specimen handling, and improved analyte stability [44]. Since the introduction of DBS sampling, microsampling devices have evolved rapidly to address the challenges of pre-analytical variability associated with DBS.

One of the most significant advancements is VAMS, which introduces a calibrated polymeric tip to control the volume of collected blood, enabling accurate fixed-volume sampling and minimizing the HCT effect [27, 28]. Other approaches to control collection volume include microfluidic-generated dried blood spot (mfDBS) sampling, quantitative dried blood spot (qDBS) sampling, absorptive paper disc (VAPD) and mini-disc (VAPDmini), and calibrated capillary-based DBS sampling [45–51]. The advancement of microfluidic devices has greatly simplified self-sampling while improving the quantification accuracy of target analytes. However, the concentration-bridging issue between plasma and blood samples in microsampling devices still needs to be addressed for the successful implementation of this promising sampling strategy in clinical care.

Because VAMS remains one of the most robust, user-friendly, and widely used microsampling technologies, the present study focused on developing a DOAC quantification method for VAMS and establishing concentration bridging with venous blood to facilitate clinical implementation. Our analytical validation demonstrated the accuracy, repeatability, reproducibility, and robustness of the VAMS and LC–MS/MS workflow. Beyond the standard validation parameters, we specifically evaluated the effects of HCT and found that only MFs were affected, whereas recoveries and accuracies remained stable. These results indicate that the quantification performance of our method is maintained across an HCT range of 20% to 60%.

Stability tests revealed significant advantages of VAMS in preserving analyte stability at RT. Whereas Opitz et al. reported that dabigatran and other DOACs were stable at RT for only 1–7 days [38], we found that all four DOACs remained stable for up to 120 days. This discrepancy might be attributable to differences in storage conditions, extraction procedures, and LC–MS/MS settings. Our findings demonstrate that, with appropriate packaging and desiccation, VAMS samples remain stable for at least 120 days, exceeding standard outpatient follow-up intervals. This stability supports the feasibility of patient-driven, remote blood collection using VAMS in clinical settings.

Although our workflow was analytically validated, several improvements could be pursued in future studies. The extraction procedure required several hours for the drying step and involved manual preparation. Potential enhancements include implementing automated sample-preparation procedures and adopting more sensitive LC-MS instrumentation to eliminate the need for drying. These modifications could substantially accelerate analysis and better support time-critical clinical decision-making.

Both previous studies and our own data consistently demonstrate that DOAC levels in plasma are generally higher than in whole blood, particularly for dabigatran, rivaroxaban, and apixaban [29, 30]. This disparity likely results from the drugs’ preferential distribution into plasma rather than inconsistencies in extraction recoveries, as also supported by DBS studies and clinical reports [30, 52–54]. To better align with prior pharmacokinetic and therapeutic monitoring studies reported with plasma concentrations, we developed conversion methods to translate DOAC concentrations measured from VAMS into corresponding plasma concentrations.

Because VAMS samples were collected via finger prick, and plasma samples from venous blood, we investigated potential differences in drug concentration between capillary and venous sources by comparing DOACs’ concentration between venous blood collected in EDTA-containing tubes and finger-prick blood. For dabigatran, rivaroxaban, and apixaban, no significant differences were found, indicating interchangeability between sample sources. However, discrepancies were observed with edoxaban, and the reason remains unclear. Possible explanations include the effects of anticoagulants in the collection tube or degradation in red blood cells due to enzymatic reactions or oxidative stress [55–58]. Nonetheless, all four DOACs exhibited strong linear relationships between venous and finger-prick samples, further supporting the interchangeability of finger-prick VAMS and venous VAMS sampling.

To derive reliable factors for converting from finger prick VAMS to plasma concentrations, we evaluated four statistical approaches: mean of ratios, ratio of means, Deming regression with zero intercept, and weighted Deming regression with zero intercept [33]. LOOCV was applied to assess model performance, and MPE, RMSPE, and MAPE were used as evaluation metrics. Among these methods, weighted Deming regression yielded the best performance. This approach is particularly appropriate because it accounts for proportional measurement errors in both the predictor and the response variable, which aligns with the nature of bioanalytical measurements [59, 60].

According to EMA/FDA bioanalytical guidelines, at least 67% of samples should demonstrate less than 20% difference between test and reference methods [61, 62]. Our method exceeded this threshold significantly, with more than 80% of samples falling within the acceptable range, demonstrating high predictive accuracy. Notably, for edoxaban, we observed a lower proportion of samples demonstrating less than 20% difference, along with unexplained discrepancies between venous and capillary sources, highlighting the need for further analytical and clinical investigations.

Fehér and Vincze et al. previously proposed a relative-recovery–based method for correcting VAMS concentrations of apixaban and rivaroxaban using measured drug concentration and HCT as input variables [26]. Their corrected VAMS concentrations also showed a systemic bias of less than 5% when compared with plasma values. However, for apixaban, their method did not meet the EMA/FDA criterion, with fewer than 60% of samples falling within ±20% of the mean of corrected VAMS and plasma concentrations. The observed difference in predictive performance is attributable to variations in study populations and sample storage conditions. Notably, their research targeted emergency care patients, whereas our cohort consisted of stable outpatients, likely contributing to more homogeneous clinical characteristics and laboratory values, especially HCT values. In addition, their study did not employ desiccants during sample storage, potentially introducing variability due to analyte degradation.

Table 5 compares the plasma-to-blood ratios obtained in this study with those reported in clinical pharmacology and biopharmaceutics reviews [52–54, 63]. Despite the ratios observed in this study being higher than those previously reported, the overall trends were consistent: dabigatran showed the highest plasma-to-blood ratio, whereas edoxaban exhibited a nearly equal distribution between plasma and blood. These patterns also align with previous DBS-based studies [29, 30].

**Table 5.** Comparison of plasma-to-blood ratios reported in clinical reports and present study.

Analyte	Plasma-to-blood ratio		Deviation (%)
	Clinical reports	Clinical samples (conversion factor)	
Dabigatran	>1.50	1.88	28.5
Rivaroxaban	1.4	1.57	12.2
Apixaban	1.25–1.43	1.64	22.2
Edoxaban	1	1.08	8.0

Although the conversion methods demonstrated acceptable performance without accounting for HCT, we further evaluated the potential impact of HCT variability by analyzing the correlation between HCT values and percentage differences between VAMS-estimated and measured plasma concentrations. Previous studies using DBS suggested that HCT exerts the greatest influence on prediction errors for dabigatran, with lesser effects observed for apixaban and rivaroxaban [30]. In our study, only dabigatran showed a statistically significant correlation between HCT and prediction error. However, despite this significance, the associated prediction errors remained less than 20%, and the coefficient of determination was modest (*R*^2^ = 0.34). Additionally, because most participants had HCT levels within the typical clinical range of 30% to 50%, any HCT-related bias is likely to be of limited clinical significance in the current cohort for all four DOACs. Collectively, these findings indicate that, although HCT may influence the estimation of plasma concentrations, further studies are required to develop a more effective equation that accounts for the effect of extreme HCT values, which may further improve safety for patients at high risk of bleeding and thrombosis.

Although our study demonstrated robust performance in converting finger-prick VAMS concentrations to corresponding plasma concentrations using LC–MS/MS analysis, the sample size was relatively small and included fewer than 40 samples, which is below the number recommended by the Clinical and Laboratory Standards Institute guideline [64]. Expanding the cohort in future studies would improve the precision of the estimated conversion factors. Further clinical validation using more independent test populations is warranted to strengthen the generalizability of these findings.

## 5. Conclusion

This study developed a VAMS method to quantify the concentrations of four DOACs (dabigatran, apixaban, rivaroxaban, and edoxaban) using LC–MS/MS. Using paired samples from 134 patients, we proposed conversion factors for estimating plasma concentrations of these four drugs and demonstrated that the estimation accuracy met EMA/FDA acceptance criteria. We anticipate that this VAMS method with LC–MS/MS could be used for DOAC TDM, supporting its integration into precision medicine strategies to prevent stroke and bleeding events, including in outpatient and remote care settings.

## Supplementary Material

Supplementary Material

## Data Availability

The data that support the findings of this study are available on request from the corresponding author, Prof. Ching-Hua Kuo. The data are not publicly available due to privacy restrictions.

## Abbreviations

CV: Coefficient of variation
CVS: Conventional venous sampling
DBS: Dried blood spot
DOAC: Direct oral anticoagulant
HCT: Hematocrit
ICH: Intracranial hemorrhage
IS: Internal standard
LLOQ: Lower limit of quantification
LOD: Limit of detection
LOOCV: Leave-one-out cross-validation
MAPE: Mean absolute percentage error
MF: Matrix factor
MPE: Mean percentage error
MS: Mass spectrometry
QC: Quality control
*R* ^2^: Coefficient of determination
rcf: Relative centrifugal force
RMSPE: Root mean squared percentage error
RT: Room temperature
SD: Standard deviation
TDM: Therapeutic drug monitoring
ULOQ: Upper limit of quantification
VAMS: Volumetric absorptive microsampling
*X^⁻^*: Expected value
