# Supplementary material for: Bridging of Finger-Prick Volumetric Absorptive Microsampling and Conventional Venous Sampling with Clinical Specimens for Direct Oral Anticoagulants Analysis by LC–MS/MS
Source: J Mass Spectrom Adv Clin Lab. 2026 Jul 8;39(1):bhyw5v67. doi: 10.21627/bhyw5v67 (PMC13750508; doi:10.21627/bhyw5v67)
Supplement: Supplementary file 1 [file jmsacl-39-1-bhyw5v67-s001.docx]

**Supplementary Material**

The following are the Supplementary data to this article:

Table S1. Accuracy for quantification of four DOACs in VAMS samples at 1500 ng mL^-1^ with 2-fold dilution.

| Diluent^a^ | Apixaban  ($\bar{\text{X}}$±SD %) | Edoxaban  ($\bar{\text{X}}$±SD %) | Dabigatran  ($\bar{\text{X}}$±SD %) | Rivaroxaban  ($\bar{\text{X}}$±SD %) |
| --- | --- | --- | --- | --- |
| Methanol (n = 5) | 97.7 ± 5.4 | 99.2 ± 8.9 | 99.1 ± 4.2 | 103.7 ± 5.5 |
| Matrix (n = 5) | 99.9 ± 4.4 | 105.0 ± 6.8 | 101.4 ± 5.5 | 105.0 ± 4.5 |

^a^ Accuracy was evaluated with 5 replicates for each diluent.

Table S2. Matrix effect for quantification of four DOACs in VAMS samples.

| Test level^a^ | Apixaban  ($\bar{\text{X}}$±SD %) | Edoxaban  ($\bar{\text{X}}$±SD %) | Dabigatran  ($\bar{\text{X}}$±SD %) | Rivaroxaban  ($\bar{\text{X}}$±SD %) |
| --- | --- | --- | --- | --- |
| Low (n = 18) | 101.7 ± 6.7 | 97.0 ± 7.6 | 100.5 ± 6.9 | 102.1 ± 7.5 |
| High (n = 18) | 97.1 ± 7.8 | 95.3 ± 5.7 | 98.2 ± 7.6 | 96.4 ± 6.0 |

^a^ Matrix effect was evaluated with 6 sources and 3 replicates/sources for each level, i.e. a total of 18 replicates

Table S3. Recovery, matrix factor and accuracy for quantification of four DOACs in VAMS samples with different hematocrit (HCT) levels.

| Analyte | HCT (%) | Recovery (n = 3)^a^  ($\bar{\text{X}}$±SD %) | |  | Matrix factor (n = 3)^a^  ($\bar{\text{X}}$±SD %) | |  | Accuracy (n = 3)^a^  ($\bar{\text{X}}$±SD %) | |
| --- | --- | --- | --- | --- | --- | --- | --- | --- | --- |
|  |  | Low^b^ | High^b^ |  | Low^b^ | High^b^ |  | Low^b^ | High^b^ |
| Dabigatran | 20 | 116.3 ± 8.6 | 95.4 ± 9.3 |  | 130.2 ± 15.2 | 138.4 ± 9.1 |  | 98.6 ± 6.4 | 94.0 ± 1.0 |
|  | 40 | 108.7 ± 8.0 | 107.6 ± 17.5 |  | 132.5 ± 15.5 | 129.2 ± 6.0 |  | 97.2 ± 6.5 | 94.1 ± 1.4 |
|  | 60 | 104.6 ± 6.5 | 110.8 ± 6.0 |  | 138.7 ± 16.1 | 141.5 ± 6.7 |  | 96.9 ± 4.6 | 92.0 ± 3.1 |
| Rivaroxaban | 20 | 112.3 ± 9.2 | 96.8 ± 6.6 |  | 100.9 ± 10.0 | 96.2 ± 5.2 |  | 98.9 ± 8.0 | 91.8 ± 1.7 |
|  | 40 | 111.1 ± 8.2 | 112.5 ± 13.0 |  | 101.2 ± 10.3 | 97.6 ± 7.9 |  | 99.4 ± 4.4 | 92.9 ± 0.8 |
|  | 60 | 107.0 ± 7.3 | 110.6 ± 6.1 |  | 110.2 ± 10.8 | 107.9 ± 4.9 |  | 100.7 ± 3.2 | 92.5 ± 3.4 |
| Apixaban | 20 | 114.9 ± 6.8 | 89.9 ± 10.1 |  | 93.2 ± 2.5 | 96.7 ± 5.8 |  | 98.9 ± 6.0 | 91.8 ± 1.2 |
|  | 40 | 106.5 ± 3.7 | 106.8 ± 19.0 |  | 93.6 ± 3.0 | 87.4 ± 3.3 |  | 96.7 ± 3.9 | 92.6 ± 0.9 |
|  | 60 | 107.6 ± 5.7 | 117.1 ± 6.0 |  | 99.7 ± 2.9 | 90.7 ± 2.9 |  | 100.9 ± 4.0 | 92.5 ± 3.9 |
| Edoxaban | 20 | 111.6 ± 16.4 | 90.6 ± 10.3 |  | 97.9 ± 9.6 | 113.0 ± 9.6 |  | 95.9 ± 9. | 92.4 ± 1.8 |
|  | 40 | 109.4 ± 10.6 | 111.3 ± 21.0 |  | 103.5 ± 10.2 | 122.0 ± 13.3 |  | 95.9 ± 4.6 | 92.2 ± 1.1 |
|  | 60 | 104.7 ± 9.5 | 102.0 ± 8.4 |  | 107.4 ± 11.5 | 135.6 ± 3.0 |  | 94.0 ± 5.5 | 90.5 ± 3.6 |

^a^ Accuracy, recovery and matrix factor were evaluated with 3 replicates for each level and sample type.

^b^ Low, 10 ng mL^-1^; High, 750 ng mL^-1^.

Figure S1. Correlation between DOAC concentrations quantified in paired finger-prick blood and venous blood samples. The solid blue line indicates the correlation line and the light green shade indicates the 95% confidence intervals (CIs). The red dashed line indicates the identity. The coefficients of determination (R^2^) and weighted Deming regression formula are also displayed on the graph.

Figure S2. Bland–Altman plots for DOAC concentrations showing the percentage differences between finger-prick blood and venous blood concentrations. The mean bias (blue line), upper and lower 95% limit of agreements (LoAs; green lines), 20% estimation error acceptance criteria (dashed red lines), and 95% confidence intervals (CIs; black error bars) are indicated.

Table S4. Weighted Deming regression analysis between VAMS and plasma concentration

| Analyte | Statistics | Estimate | SE^a^ | 95% CI^a^ |
| --- | --- | --- | --- | --- |
| Dabigatran | Intercept | -4.429 | 3.913 | -12.44−3.586 |
|  | Slope | 1.957 | 0.073 | 1.808−2.106 |
| Rivaroxaban | Intercept | 0.408 | 1.370 | -2.386−3.203 |
|  | Slope | 1.559 | 0.042 | 1.473−1.645 |
| Apixaban | Intercept | -4.824 | 4.880 | -14.75−5.105 |
|  | Slope | 1.709 | 0.070 | 1.567−1.861 |
| Edoxaban | Intercept | -0.148 | 0.578 | -1.322−1.027 |
|  | Slope | 1.087 | 0.039 | 1.008−1.165 |

^a^ SE, standard error; CI, confidence interval.

Table S5. LOOCV of four methods to translate VAMS concentration to plasma concentration.

| Analyte | Method | MPE^a^ (%) | RMSPE^a^ (%) | MAPE^a^ (%) |
| --- | --- | --- | --- | --- |
| Dabigatran | Mean of ratios | 0.77 | 12.82 | 10.17 |
|  | Ratio of means | 0.55 | 12.81 | 10.23 |
|  | Deming^b^ | -0.91 | 12.91 | 10.55 |
|  | Weighted Deming^b,c^ | 0.00 | 12.81 | 10.28 |
| Rivaroxaban | Mean of ratios | 1.08 | 14.42 | 10.66 |
|  | Ratio of means | -4.65 | 14.82 | 9.97 |
|  | Deming^b^ | -7.34 | 15.75 | 10.70 |
|  | Weighted Deming^b,c^ | 0.00 | 14.32 | 10.34 |
| Apixaban | Mean of ratios | 0.78 | 12.73 | 10.16 |
|  | Ratio of means | 3.29 | 13.18 | 10.78 |
|  | Deming^b^ | 4.17 | 13.74 | 11.09 |
|  | Weighted Deming^b,c^ | 0.00 | 12.70 | 10.05 |
| Edoxaban | Mean of ratios | 1.24 | 16.13 | 12.87 |
|  | Ratio of means | 2.88 | 16.33 | 13.10 |
|  | Deming^b^ | 6.36 | 17.29 | 14.23 |
|  | Weighted Deming^b,c^ | 0.00 | 16.07 | 12.84 |

^a^ MPE, mean percentage error; RMSPE, root mean squared percentage error; MAPE, mean absolute percentage error.

^b^ Intercepts are not fitted, and fixed at zero.

^c^ Assume error variances are proportional to the squares of true values.

Table S6. Conversion factors, mean percentage errors (MPEs), root mean squared percentage errors (RMSPEs), and mean absolute percentage errors (MAPEs) of the conversion methods.

|  | Dabigatran | Rivaroxaban | Apixaban | Edoxaban |
| --- | --- | --- | --- | --- |
| Conversion factor | 1.88 | 1.57 | 1.64 | 1.08 |
| MPE | 0.0% | 0.0% | 0.0% | 0.0% |
| RMSPE | 12.4% | 13.9% | 12.3% | 15.6% |
| MAPE | 9.9% | 10.0% | 9.8% | 12.5% |

Figure S3. Correlation between percentage difference of measured and estimated plasma concentration, and hematocrit (HCT). Light green regions are 95% confidence intervals (CIs). Ordinary linear regression formula, the coefficients of determination (R^2^) and p value of slope are indicated.
